# P-2191. Trends in Influenza Vaccine Disparities in the U.S. Following the Introduction of COVID-19: Insights from the National Health Interview Survey

**DOI:** 10.1093/ofid/ofaf695.2354

**Published:** 2026-01-11

**Authors:** Margaret L Lind, Emily Goldmann, Gregory Lalonde

**Affiliations:** Boston University School of Public Health, Boston, Massachusetts; Boston University, Boston, Massachusetts; University of Maryland, Baltimore, Maryland

## Abstract

**Background:**

Influenza vaccination coverage in the United States remains below 50%. Historical racial and ethnic disparities in coverage have been previously documented, but recent trends suggest shifts these disparities. NH-Black individuals, a historically under-vaccinated group, have experienced consistent increases in coverage over the past few seasons while data suggests that historically better vaccinated groups, such as NH-, have experienced declining coverage rates. In this study, we examine racial and ethnic disparities in influenza vaccine coverage since the onset of the COVID-19 pandemic, assessing whether these disparities persisted after stratification by likely drivers of coverage including age, healthcare worker status, underlying health conditions, and access to care.

**Figure 1.**
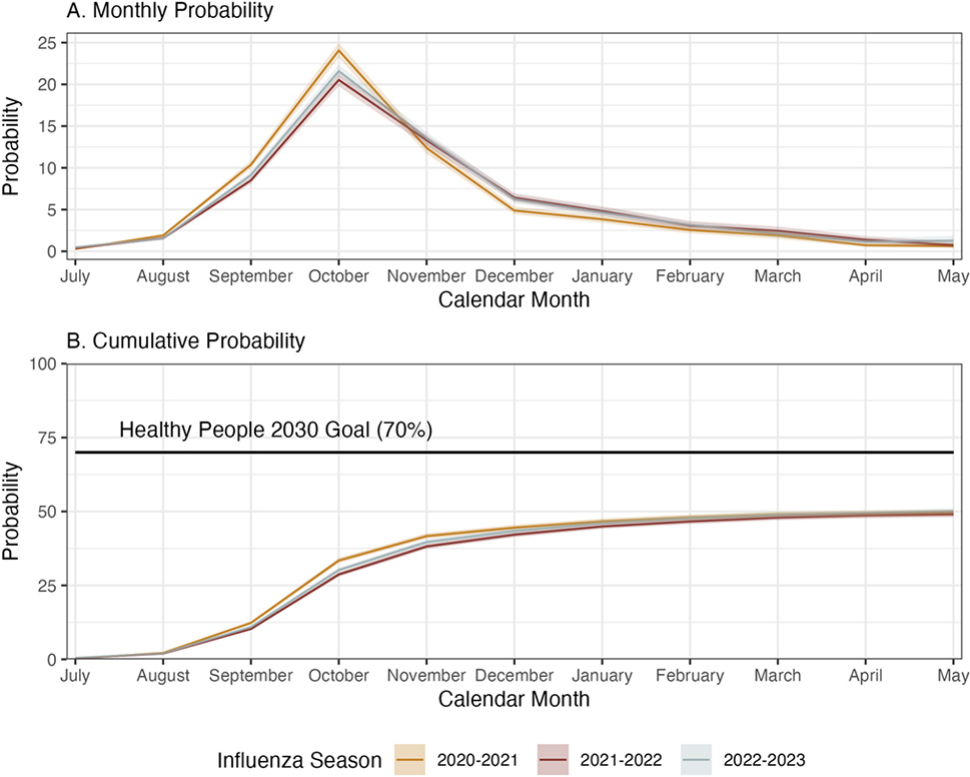
Influenza Vaccination Probability for the 2020-2021 through 2022-2023 Influenza Seasons

**Figure 2.**
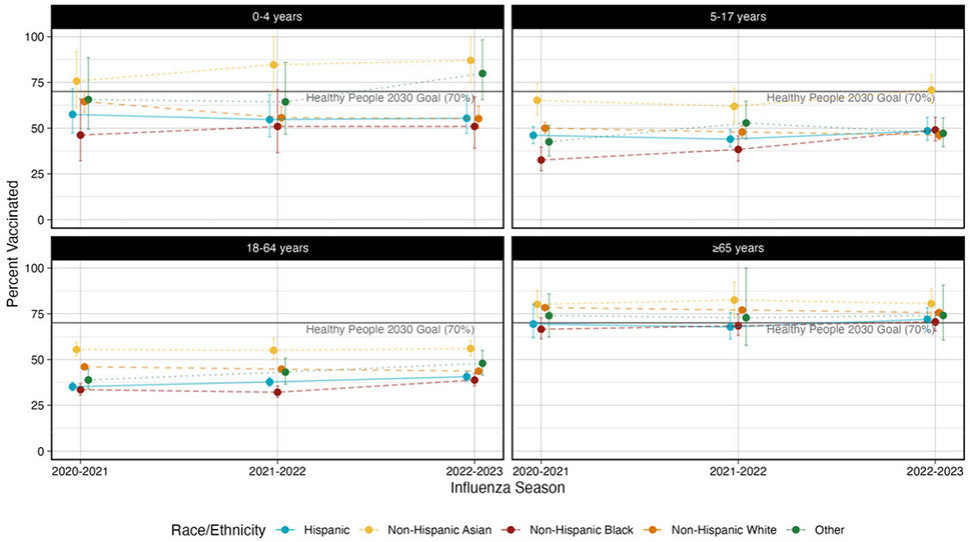
Influenza Vaccination Coverage by Age and Race/Ethnicity for the 2020-2021 through 2022-2023 Influenza Seasons

**Methods:**

We estimated influenza vaccine coverage using 2020-2023 National Health Interview Survey data collected between August and June. Coverage was estimated via Kaplan Meier analysis and self-reported vaccination between July and May in the prior 12 months.

**Figure 3.**
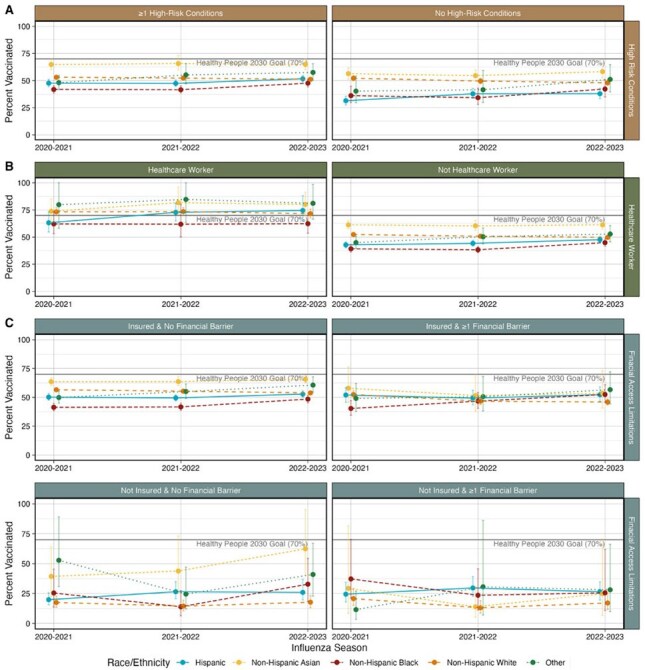
Influenza Vaccination Coverage by Race/Ethnicity and High-Risk Status for the 2020-2021 through 2022-2023 Influenza Seasons

**Results:**

Among the 106,126 people included in the analysis, vaccination coverage ranged between 49.1 (CI: 48.2-50.0%) in 2021-2022 to 50.1% (CI: 49.2-51.0%) in 2022-2023. People who identified as NH-Black had the lowest coverage for all three seasons but experienced the largest proportional increase in coverage (20.1% [CI: 8.5-31.6%]). Coverage declined among people who identified as NH-White by 3 (CI: 1-4) percentage points, with the largest decreases among children aged 0-4 (9 [CI: 0-15] percentage points) and insured people with financial barriers to healthcare (7 [CI: 2-11] percentage points).

**Conclusion:**

Despites notable gains among people who identified as NH-Black, disparities persisted and, as of the 2022-2023 season, coverage among this population remained 25.1 percentage points below the Healthy People 2030 goal of 70%. While all other racial and ethnic groups saw increases in coverage, people who identified as NH-White experienced declines, particularly young children and insured individuals with financial barriers. These findings suggest that vaccine uptake is nuanced and that, addressing financial barriers and targeted messaging may help improve uptake.

**Disclosures:**

All Authors: No reported disclosures

